# 3,5-Bis(4-fluoro­phen­yl)-1-(4-nitro­phen­yl)-4,5-dihydro-1*H*-pyrazole

**DOI:** 10.1107/S160053681204370X

**Published:** 2012-10-27

**Authors:** Seranthimata Samshuddin, Badiadka Narayana, Hemmige S. Yathirajan, Thomas Gerber, Eric Hosten, Richard Betz

**Affiliations:** a Mangalore University, Department of Studies in Chemistry, Mangalagangotri 574 199, India; b University of Mysore, Department of Studies in Chemistry, Manasagangotri, Mysore 570 006, India; c Nelson Mandela Metropolitan University, Summerstrand Campus, Department of Chemistry, University Way, Summerstrand, PO Box 77000, Port Elizabeth, 6031, South Africa

## Abstract

In the title compound, C_21_H_15_F_2_N_3_O_2_, a pyrazole derivative bearing three aromatic substituents, the central five-membered heterocyclic ring makes dihedral angles of 1.77 (14), 3.68 (13) and 72.15 (14)° with the three benzene rings. In the crystal, C—H⋯O and C—H⋯F inter­actions connect the mol­ecules into double layers parallel to the *bc* plane.

## Related literature

For general information about the pharmacological properties and medical applications of pyrazole derivatives, see: Kumar *et al.* (2009 ▶); Sarojini *et al.* (2010 ▶); Samshuddin *et al.* (2012 ▶). For the crystal structures of other pyrazole derivatives, see: Baktır *et al.* (2011 ▶); Jasinski *et al.* (2012 ▶). For the puckering analysis of cyclic motifs, see: Cremer & Pople (1975 ▶). For graph-set analysis of hydrogen bonds, see: Etter *et al.* (1990 ▶); Bernstein *et al.* (1995 ▶).
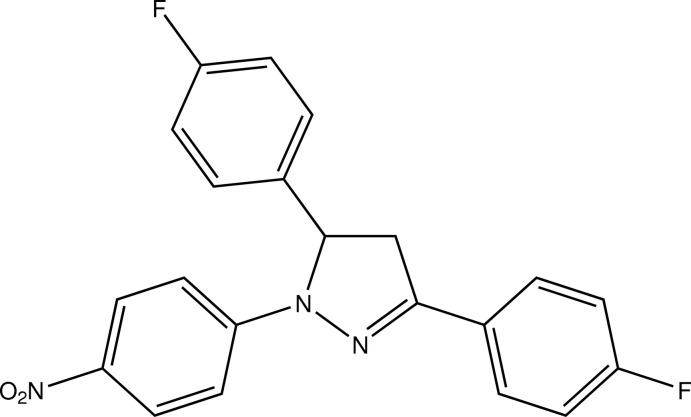


## Experimental

### 

#### Crystal data

C_21_H_15_F_2_N_3_O_2_
*M* *_r_* = 379.36
Monoclinic, 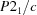

*a* = 13.2884 (13) Å
*b* = 12.7364 (10) Å
*c* = 11.4656 (9) Å
β = 115.324 (3)°
*V* = 1754.0 (3) Å^3^
*Z* = 4
Mo *K*α radiation
μ = 0.11 mm^−1^
*T* = 200 K
0.57 × 0.33 × 0.27 mm

#### Data collection

Bruker APEXII CCD diffractometer
Absorption correction: multi-scan (*SADABS*; Bruker, 2008 ▶) *T*_min_ = 0.692, *T*_max_ = 0.971
15973 measured reflections
4301 independent reflections
3066 reflections with *I* > 2σ(*I*)
*R*_int_ = 0.052

#### Refinement

*R*[*F* ^2^ > 2σ(*F* ^2^)] = 0.067
*wR*(*F* ^2^) = 0.221
*S* = 1.06
4301 reflections
253 parameters
H-atom parameters constrained
Δρ_max_ = 0.41 e Å^−3^
Δρ_min_ = −0.39 e Å^−3^

### 

Data collection: *APEX2* (Bruker, 2010 ▶); cell refinement: *SAINT* (Bruker, 2010 ▶); data reduction: *SAINT*; program(s) used to solve structure: *SHELXS97* (Sheldrick, 2008 ▶); program(s) used to refine structure: *SHELXL97* (Sheldrick, 2008 ▶); molecular graphics: *ORTEP-3* (Farrugia, 1997 ▶) and *Mercury* (Macrae *et al.*, 2008 ▶); software used to prepare material for publication: *SHELXL97* and *PLATON* (Spek, 2009 ▶).

## Supplementary Material

Crystal structure: contains datablock(s) I, global. DOI: 10.1107/S160053681204370X/is5210sup1.cif

Supplementary material file. DOI: 10.1107/S160053681204370X/is5210Isup2.cdx

Structure factors: contains datablock(s) I. DOI: 10.1107/S160053681204370X/is5210Isup3.hkl

Supplementary material file. DOI: 10.1107/S160053681204370X/is5210Isup4.cml

Additional supplementary materials:  crystallographic information; 3D view; checkCIF report

## Figures and Tables

**Table 1:** Hydrogen-bond geometry (Å, °)

*D*—H⋯*A*	*D*—H	H⋯*A*	*D*⋯*A*	*D*—H⋯*A*
C12—H12⋯O2^i^	0.95	2.41	3.305 (3)	157
C16—H16⋯F2^ii^	0.95	2.55	3.427 (3)	154
C26—H26⋯F1^iii^	0.95	2.56	3.494 (3)	169

Symmetry codes: (i) 
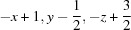
; (ii) 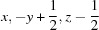; (iii) 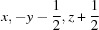.

**Fig. 1.**
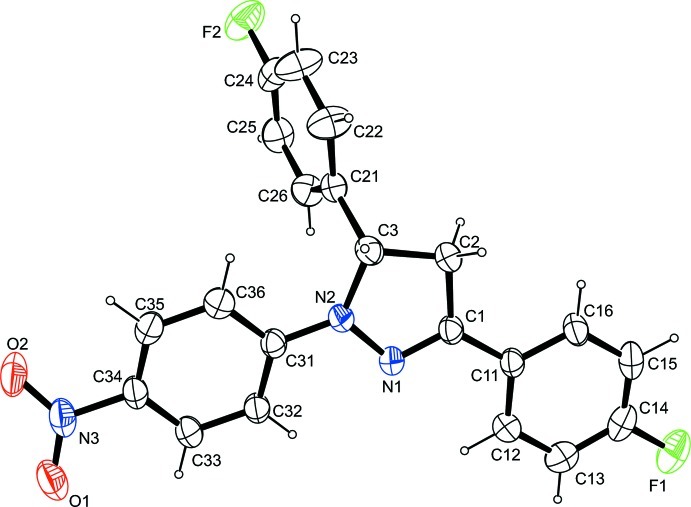
The molecular structure of the title compound, with atom labels and anisotropic displacement ellipsoids (drawn at 50% probability level).

**Fig. 2.**
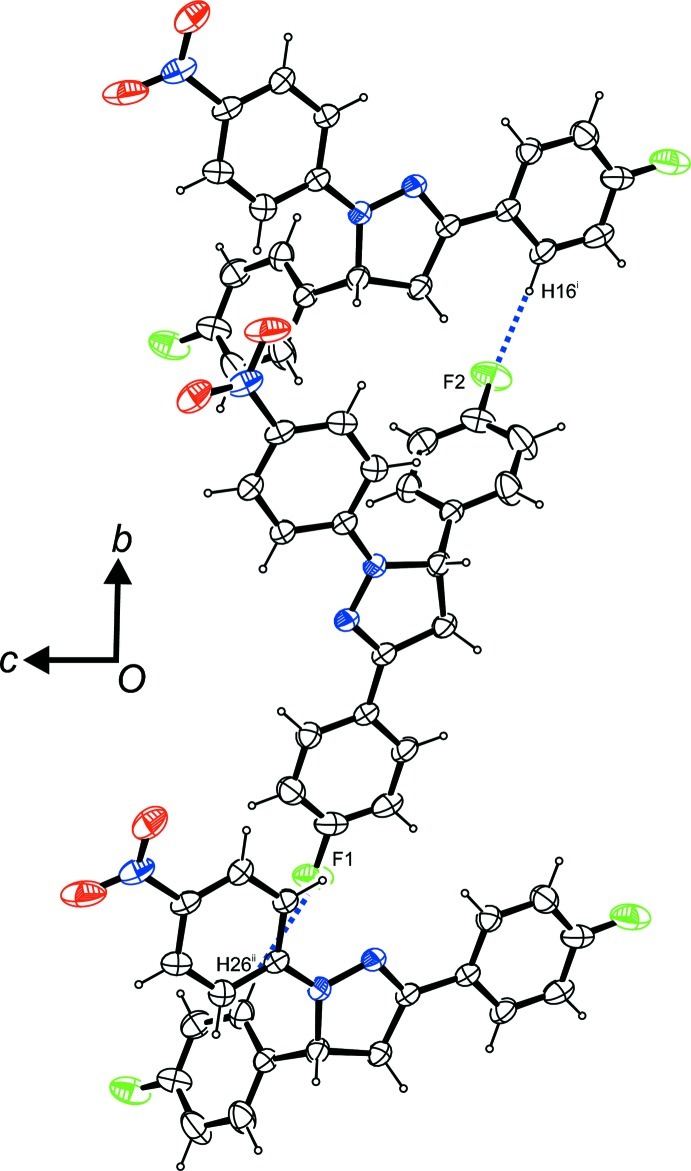
Intermolecular contacts, viewed along [-1 0 0]. For clarity, only an arbitrary selection of intermolecular contacts is shown. [Symmetry codes: (i) x, -y + 1/2, z + 1/2; (ii) x, -y - 1/2, z - 1/2].

**Fig. 3.**
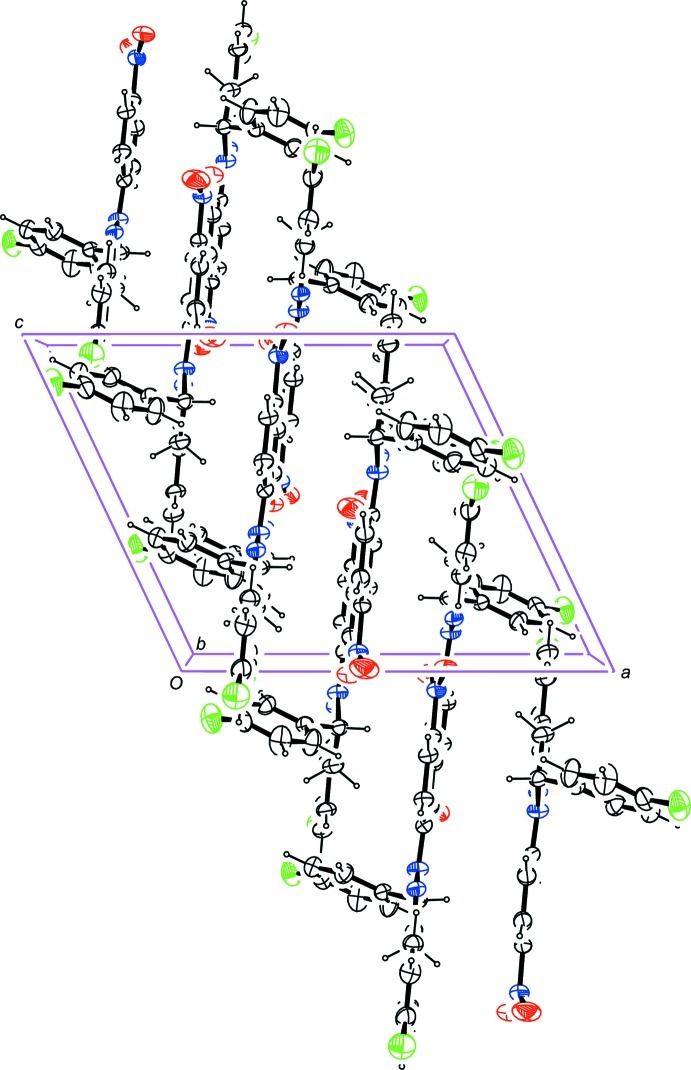
Molecular packing of the title compound, viewed along [0 1 0] (anisotropic displacement ellipsoids drawn at 50% probability level).

## supplementary crystallographic information

### Comment

Pyrazole derivatives are well known for their broad spectrum of pharmacological properties and have been found – among others – to exhibit antimicrobial, antioxidant, antiamoebic, anti-inflammatory, analgesic, antidepressant and anticancer activity (Kumar *et al.*, 2009; Sarojini *et al.*, 2010; Samshuddin *et al.*, 2012). Because of these various interesting fields of application as well as their fairly assessable path of synthesis, the pyrazoline ring became a center of attraction for organic chemists. The crystal structures of some pyrazolines derived from 4,4'-difluoro chalcone have been reported (Baktır *et al.*, 2011; Jasinski *et al.*, 2012). Fuelled by our ongoing interest in pharmacological active compounds, the title compound was synthesized.

Three phenyl-derived substituents are bonded to a central 4,5-dihydro-1*H*-pyrazole moiety. The least-squares planes defined by the C11–C16, C31–C36 and C21–C26 benzene rings enclose dihedral angles of 1.77 (14), 3.68 (13) and 72.15 (14)°, respectively, with the least-squares plane defined by the intracyclic atoms of the central five-membered heterocycle with the largest angle formed by one of the two *para*-fluoro phenyl groups. A conformational analysis of the 4,5-dihydro-1*H*-pyrazole moiety is precluded due to its low puckering amplitude (Cremer & Pople, 1975). The nitro group is slightly tilted out of plane of the least-square plane defined by the carbon atoms of the aromatic moiety it is bonded to, the corresponding O2—N3—C34—C35 torsion angle being 17.0 (3)° (Fig. 1).

In the crystal, C—H···O and C—H···F contacts can be observed whose range falls by more than 0.1 Å below the sum of van-der-Waals radii of the atoms participating in them. These are exclusively supported by hydrogen atoms bonded to *para*-fluoro phenyl groups. Metrical parameters as well as information about the symmetry of these contacts are summarized in Table 1. In terms of graph-set analysis (Etter *et al.*, 1990; Bernstein *et al.*, 1995), the descriptor for the C—H···O contacts is *C*(12) on the unary level, while the C—H···F contacts necessitate a *C*(11)*C*(11) descriptor on the same level. In total, the molecules are connected to double layers parallel to the *bc* plane. The shortest intercentroid distance between two aromatic systems was measured at 4.8923 (17) Å and is observed between the two different fluorinated phenyl groups in neighbouring molecules. Taking into account the centroid of the 4,5-dihydro-1*H*-pyrazole moiety as well, the shortest intercentroid distance is found at 3.5918 (15) Å between this pyrazole unit and the nitrated phenyl group (Fig. 2). The packing of the title compound in the crystal structure is shown in Figure 3.

### Experimental

A mixture of 4,4'-difluoro chalcone (2.68 g, 0.01 mol) and 4-nitrophenyl hydrazine (1.53 g, 0.01 mol) was refluxed in glacial acetic acid (50 ml) for 6 h. The reaction mixture was cooled and pourred into ice-cold water (50 ml). The precipitate was collected by filtration and purified by recrystallization from ethanol (yield: 74%). Yellow blocks, suitable for the X-ray diffraction study, were grown from a DMF solution by slow evaporation at room temperature.

### Refinement

H atoms were placed in calculated positions (C—H 0.95 Å for aromatic carbon atoms, C—H 0.99 Å for the methylene group and C—H 1.00 Å for the methine group) and were included in the refinement in the riding model approximation, with *U*(H) set to 1.2*U*_eq_(C).

### Crystal data

**Table:**

C_21_H_15_F_2_N_3_O_2_	*F*(000) = 784
*M**_r_* = 379.36	*D*_x_ = 1.437 Mg m^−^^3^
Monoclinic, *P*2_1_/*c*	Melting point: 443 K
Hall symbol: -P 2ybc	Mo *K*α radiation, λ = 0.71073 Å
*a* = 13.2884 (13) Å	Cell parameters from 6702 reflections
*b* = 12.7364 (10) Å	θ = 2.3–27.9°
*c* = 11.4656 (9) Å	µ = 0.11 mm^−^^1^
β = 115.324 (3)°	*T* = 200 K
*V* = 1754.0 (3) Å^3^	Block, orange
*Z* = 4	0.57 × 0.33 × 0.27 mm

### Data collection

**Table:**

Bruker APEXII CCD diffractometer	4301 independent reflections
Radiation source: fine-focus sealed tube	3066 reflections with *I* > 2σ(*I*)
Graphite monochromator	*R*_int_ = 0.052
φ and ω scans	θ_max_ = 28.3°, θ_min_ = 2.3°
Absorption correction: multi-scan (*SADABS*; Bruker, 2008)	*h* = −17→17
*T*_min_ = 0.692, *T*_max_ = 0.971	*k* = −15→16
15973 measured reflections	*l* = −15→14

### Refinement

**Table:**

Refinement on *F*^2^	Primary atom site location: structure-invariant direct methods
Least-squares matrix: full	Secondary atom site location: difference Fourier map
*R*[*F*^2^ > 2σ(*F*^2^)] = 0.067	Hydrogen site location: inferred from neighbouring sites
*wR*(*F*^2^) = 0.221	H-atom parameters constrained
*S* = 1.06	*w* = 1/[σ^2^(*F*_o_^2^) + (0.1331*P*)^2^ + 0.5062*P*] where *P* = (*F*_o_^2^ + 2*F*_c_^2^)/3
4301 reflections	(Δ/σ)_max_ < 0.001
253 parameters	Δρ_max_ = 0.41 e Å^−^^3^
0 restraints	Δρ_min_ = −0.39 e Å^−^^3^

### Fractional atomic coordinates and isotropic or equivalent isotropic displacement parameters (Å^2^)

**Table:**

	*x*	*y*	*z*	*U*_iso_*/*U*_eq_	
F1	0.11689 (15)	−0.42461 (13)	−0.04507 (17)	0.0638 (5)	
F2	0.01460 (13)	0.43563 (13)	0.35591 (18)	0.0611 (5)	
O1	0.61528 (16)	0.02802 (17)	1.00769 (16)	0.0536 (5)	
O2	0.57223 (18)	0.19339 (18)	0.98021 (18)	0.0663 (6)	
N1	0.30617 (15)	−0.05788 (13)	0.36124 (16)	0.0305 (4)	
N2	0.32892 (15)	0.04326 (14)	0.40815 (16)	0.0327 (4)	
N3	0.56940 (16)	0.10368 (18)	0.93869 (18)	0.0428 (5)	
C1	0.25491 (17)	−0.05414 (16)	0.23711 (18)	0.0297 (4)	
C2	0.2351 (2)	0.05569 (17)	0.1837 (2)	0.0368 (5)	
H2A	0.1545	0.0717	0.1396	0.044*	
H2B	0.2688	0.0663	0.1225	0.044*	
C3	0.29364 (18)	0.12390 (17)	0.30617 (19)	0.0322 (5)	
H3	0.3607	0.1583	0.3047	0.039*	
C11	0.21963 (17)	−0.15094 (17)	0.16168 (18)	0.0298 (4)	
C12	0.24429 (19)	−0.24829 (18)	0.2240 (2)	0.0354 (5)	
H12	0.2849	−0.2511	0.3151	0.042*	
C13	0.2103 (2)	−0.34014 (19)	0.1545 (3)	0.0426 (5)	
H13	0.2270	−0.4063	0.1968	0.051*	
C14	0.1515 (2)	−0.3339 (2)	0.0222 (2)	0.0431 (6)	
C15	0.1269 (2)	−0.2404 (2)	−0.0425 (2)	0.0437 (6)	
H15	0.0871	−0.2385	−0.1338	0.052*	
C16	0.16125 (19)	−0.14838 (19)	0.0279 (2)	0.0375 (5)	
H16	0.1448	−0.0827	−0.0156	0.045*	
C21	0.21828 (17)	0.20653 (17)	0.32160 (18)	0.0303 (4)	
C22	0.2177 (2)	0.30641 (19)	0.2750 (3)	0.0495 (6)	
H22	0.2653	0.3225	0.2350	0.059*	
C23	0.1486 (3)	0.3840 (2)	0.2857 (3)	0.0570 (7)	
H23	0.1479	0.4525	0.2528	0.068*	
C24	0.08164 (19)	0.35925 (19)	0.3447 (3)	0.0422 (6)	
C25	0.0790 (2)	0.2616 (2)	0.3912 (2)	0.0431 (6)	
H25	0.0308	0.2464	0.4309	0.052*	
C26	0.14779 (19)	0.18489 (19)	0.3796 (2)	0.0390 (5)	
H26	0.1468	0.1163	0.4117	0.047*	
C31	0.39135 (16)	0.05788 (16)	0.53770 (18)	0.0289 (4)	
C32	0.42036 (18)	−0.02827 (17)	0.62266 (19)	0.0321 (4)	
H32	0.3995	−0.0972	0.5896	0.039*	
C33	0.47889 (18)	−0.01300 (18)	0.7534 (2)	0.0344 (5)	
H33	0.4983	−0.0711	0.8108	0.041*	
C34	0.50939 (17)	0.08796 (18)	0.80077 (19)	0.0331 (5)	
C35	0.48391 (18)	0.17349 (18)	0.7191 (2)	0.0348 (5)	
H35	0.5063	0.2419	0.7534	0.042*	
C36	0.42597 (18)	0.15954 (18)	0.5877 (2)	0.0346 (5)	
H36	0.4095	0.2181	0.5311	0.042*	

### Atomic displacement parameters (Å^2^)

**Table:**

	*U*^11^	*U*^22^	*U*^33^	*U*^12^	*U*^13^	*U*^23^
F1	0.0698 (11)	0.0542 (10)	0.0696 (11)	−0.0138 (8)	0.0319 (9)	−0.0309 (8)
F2	0.0490 (9)	0.0516 (10)	0.0853 (12)	0.0049 (7)	0.0312 (9)	−0.0195 (8)
O1	0.0509 (10)	0.0741 (14)	0.0274 (8)	0.0024 (9)	0.0089 (7)	−0.0019 (8)
O2	0.0681 (13)	0.0731 (14)	0.0428 (10)	0.0056 (11)	0.0096 (9)	−0.0301 (10)
N1	0.0346 (9)	0.0314 (9)	0.0248 (8)	0.0006 (7)	0.0119 (7)	−0.0021 (7)
N2	0.0425 (10)	0.0293 (9)	0.0234 (8)	0.0018 (7)	0.0113 (7)	0.0009 (7)
N3	0.0354 (10)	0.0617 (14)	0.0292 (9)	−0.0014 (9)	0.0117 (8)	−0.0124 (9)
C1	0.0316 (10)	0.0352 (11)	0.0239 (9)	0.0015 (8)	0.0133 (8)	−0.0014 (8)
C2	0.0474 (13)	0.0379 (12)	0.0248 (9)	0.0063 (10)	0.0151 (9)	0.0025 (8)
C3	0.0356 (11)	0.0350 (11)	0.0267 (9)	0.0027 (8)	0.0139 (8)	0.0035 (8)
C11	0.0301 (10)	0.0366 (11)	0.0233 (9)	0.0008 (8)	0.0121 (8)	−0.0025 (8)
C12	0.0364 (11)	0.0373 (11)	0.0308 (10)	0.0015 (9)	0.0127 (9)	0.0014 (9)
C13	0.0449 (13)	0.0362 (12)	0.0505 (14)	0.0009 (10)	0.0240 (11)	−0.0002 (10)
C14	0.0418 (13)	0.0435 (13)	0.0490 (13)	−0.0072 (10)	0.0243 (11)	−0.0179 (11)
C15	0.0465 (14)	0.0559 (15)	0.0288 (10)	−0.0052 (11)	0.0162 (10)	−0.0114 (10)
C16	0.0428 (12)	0.0456 (13)	0.0233 (9)	0.0011 (10)	0.0132 (9)	−0.0011 (9)
C21	0.0319 (10)	0.0334 (11)	0.0261 (9)	−0.0009 (8)	0.0128 (8)	−0.0010 (8)
C22	0.0518 (15)	0.0375 (13)	0.0737 (18)	0.0024 (11)	0.0408 (14)	0.0105 (12)
C23	0.0619 (17)	0.0300 (13)	0.091 (2)	0.0033 (12)	0.0439 (16)	0.0080 (13)
C24	0.0311 (11)	0.0396 (13)	0.0511 (14)	0.0004 (9)	0.0132 (10)	−0.0151 (10)
C25	0.0375 (12)	0.0548 (15)	0.0420 (12)	0.0000 (10)	0.0217 (10)	−0.0030 (11)
C26	0.0415 (12)	0.0412 (12)	0.0392 (11)	−0.0023 (10)	0.0221 (10)	0.0042 (9)
C31	0.0287 (10)	0.0344 (11)	0.0251 (9)	0.0009 (8)	0.0128 (8)	−0.0020 (8)
C32	0.0363 (11)	0.0330 (11)	0.0255 (9)	−0.0020 (8)	0.0118 (8)	−0.0023 (8)
C33	0.0369 (11)	0.0404 (12)	0.0256 (9)	−0.0004 (9)	0.0132 (8)	0.0004 (8)
C34	0.0273 (10)	0.0460 (12)	0.0244 (9)	0.0007 (9)	0.0094 (8)	−0.0073 (9)
C35	0.0317 (11)	0.0355 (11)	0.0376 (11)	−0.0023 (9)	0.0151 (9)	−0.0097 (9)
C36	0.0332 (11)	0.0354 (11)	0.0355 (11)	−0.0003 (9)	0.0150 (9)	−0.0007 (9)

### Geometric parameters (Å, º)

**Table:**

F1—C14	1.356 (3)	C15—C16	1.385 (3)
F2—C24	1.362 (3)	C15—H15	0.9500
O1—N3	1.230 (3)	C16—H16	0.9500
O2—N3	1.232 (3)	C21—C22	1.378 (3)
N1—C1	1.289 (2)	C21—C26	1.388 (3)
N1—N2	1.379 (2)	C22—C23	1.389 (4)
N2—C31	1.369 (2)	C22—H22	0.9500
N2—C3	1.474 (3)	C23—C24	1.364 (4)
N3—C34	1.448 (3)	C23—H23	0.9500
C1—C11	1.463 (3)	C24—C25	1.360 (4)
C1—C2	1.504 (3)	C25—C26	1.383 (3)
C2—C3	1.548 (3)	C25—H25	0.9500
C2—H2A	0.9900	C26—H26	0.9500
C2—H2B	0.9900	C31—C32	1.407 (3)
C3—C21	1.514 (3)	C31—C36	1.411 (3)
C3—H3	1.0000	C32—C33	1.376 (3)
C11—C16	1.392 (3)	C32—H32	0.9500
C11—C12	1.398 (3)	C33—C34	1.387 (3)
C12—C13	1.378 (3)	C33—H33	0.9500
C12—H12	0.9500	C34—C35	1.381 (3)
C13—C14	1.380 (4)	C35—C36	1.379 (3)
C13—H13	0.9500	C35—H35	0.9500
C14—C15	1.367 (4)	C36—H36	0.9500
			
C1—N1—N2	108.68 (16)	C15—C16—H16	119.6
C31—N2—N1	118.70 (16)	C11—C16—H16	119.6
C31—N2—C3	127.24 (18)	C22—C21—C26	118.4 (2)
N1—N2—C3	113.53 (16)	C22—C21—C3	119.40 (18)
O1—N3—O2	123.6 (2)	C26—C21—C3	122.22 (19)
O1—N3—C34	118.9 (2)	C21—C22—C23	121.1 (2)
O2—N3—C34	117.5 (2)	C21—C22—H22	119.4
N1—C1—C11	120.38 (18)	C23—C22—H22	119.4
N1—C1—C2	113.68 (18)	C24—C23—C22	118.4 (2)
C11—C1—C2	125.93 (17)	C24—C23—H23	120.8
C1—C2—C3	102.70 (16)	C22—C23—H23	120.8
C1—C2—H2A	111.2	C25—C24—F2	119.2 (2)
C3—C2—H2A	111.2	C25—C24—C23	122.5 (2)
C1—C2—H2B	111.2	F2—C24—C23	118.3 (2)
C3—C2—H2B	111.2	C24—C25—C26	118.6 (2)
H2A—C2—H2B	109.1	C24—C25—H25	120.7
N2—C3—C21	113.17 (16)	C26—C25—H25	120.7
N2—C3—C2	101.21 (16)	C25—C26—C21	121.0 (2)
C21—C3—C2	113.27 (18)	C25—C26—H26	119.5
N2—C3—H3	109.6	C21—C26—H26	119.5
C21—C3—H3	109.6	N2—C31—C32	120.28 (18)
C2—C3—H3	109.6	N2—C31—C36	120.42 (19)
C16—C11—C12	118.82 (19)	C32—C31—C36	119.30 (19)
C16—C11—C1	121.22 (19)	C33—C32—C31	120.3 (2)
C12—C11—C1	119.97 (18)	C33—C32—H32	119.9
C13—C12—C11	120.7 (2)	C31—C32—H32	119.9
C13—C12—H12	119.7	C32—C33—C34	119.5 (2)
C11—C12—H12	119.7	C32—C33—H33	120.3
C12—C13—C14	118.5 (2)	C34—C33—H33	120.3
C12—C13—H13	120.7	C35—C34—C33	121.33 (19)
C14—C13—H13	120.7	C35—C34—N3	119.5 (2)
F1—C14—C15	119.3 (2)	C33—C34—N3	119.2 (2)
F1—C14—C13	118.1 (2)	C36—C35—C34	120.0 (2)
C15—C14—C13	122.6 (2)	C36—C35—H35	120.0
C14—C15—C16	118.6 (2)	C34—C35—H35	120.0
C14—C15—H15	120.7	C35—C36—C31	119.6 (2)
C16—C15—H15	120.7	C35—C36—H36	120.2
C15—C16—C11	120.8 (2)	C31—C36—H36	120.2
			
C1—N1—N2—C31	−174.73 (17)	C2—C3—C21—C26	85.0 (2)
C1—N1—N2—C3	−2.4 (2)	C26—C21—C22—C23	0.1 (4)
N2—N1—C1—C11	−179.48 (17)	C3—C21—C22—C23	179.1 (3)
N2—N1—C1—C2	−0.7 (2)	C21—C22—C23—C24	0.6 (5)
N1—C1—C2—C3	3.2 (2)	C22—C23—C24—C25	−1.0 (4)
C11—C1—C2—C3	−178.07 (19)	C22—C23—C24—F2	179.5 (3)
C31—N2—C3—C21	−62.7 (3)	F2—C24—C25—C26	−179.8 (2)
N1—N2—C3—C21	125.73 (19)	C23—C24—C25—C26	0.8 (4)
C31—N2—C3—C2	175.7 (2)	C24—C25—C26—C21	0.0 (4)
N1—N2—C3—C2	4.2 (2)	C22—C21—C26—C25	−0.4 (3)
C1—C2—C3—N2	−4.1 (2)	C3—C21—C26—C25	−179.3 (2)
C1—C2—C3—C21	−125.55 (18)	N1—N2—C31—C32	−7.3 (3)
N1—C1—C11—C16	178.05 (19)	C3—N2—C31—C32	−178.48 (19)
C2—C1—C11—C16	−0.6 (3)	N1—N2—C31—C36	173.54 (18)
N1—C1—C11—C12	−1.8 (3)	C3—N2—C31—C36	2.4 (3)
C2—C1—C11—C12	179.6 (2)	N2—C31—C32—C33	−177.13 (19)
C16—C11—C12—C13	−0.8 (3)	C36—C31—C32—C33	2.0 (3)
C1—C11—C12—C13	179.1 (2)	C31—C32—C33—C34	−0.2 (3)
C11—C12—C13—C14	0.0 (3)	C32—C33—C34—C35	−1.2 (3)
C12—C13—C14—F1	−179.0 (2)	C32—C33—C34—N3	178.99 (18)
C12—C13—C14—C15	0.8 (4)	O1—N3—C34—C35	−162.4 (2)
F1—C14—C15—C16	178.9 (2)	O2—N3—C34—C35	17.0 (3)
C13—C14—C15—C16	−0.9 (4)	O1—N3—C34—C33	17.4 (3)
C14—C15—C16—C11	0.1 (3)	O2—N3—C34—C33	−163.2 (2)
C12—C11—C16—C15	0.7 (3)	C33—C34—C35—C36	0.7 (3)
C1—C11—C16—C15	−179.1 (2)	N3—C34—C35—C36	−179.45 (18)
N2—C3—C21—C22	151.6 (2)	C34—C35—C36—C31	1.1 (3)
C2—C3—C21—C22	−94.0 (3)	N2—C31—C36—C35	176.68 (18)
N2—C3—C21—C26	−29.5 (3)	C32—C31—C36—C35	−2.4 (3)

### Hydrogen-bond geometry (Å, º)

**Table:**

*D*—H···*A*	*D*—H	H···*A*	*D*···*A*	*D*—H···*A*
C12—H12···O2^i^	0.95	2.41	3.305 (3)	157
C16—H16···F2^ii^	0.95	2.55	3.427 (3)	154
C26—H26···F1^iii^	0.95	2.56	3.494 (3)	169

Symmetry codes: (i) −*x*+1, *y*−1/2, −*z*+3/2; (ii) *x*, −*y*+1/2, *z*−1/2; (iii) *x*, −*y*−1/2, *z*+1/2.
